# Outcomes of Group D Retinoblastoma With Resistant Vitreous Seeds After Integration of Intravitreal Chemotherapy to the Treatment Protocol

**DOI:** 10.7759/cureus.11757

**Published:** 2020-11-28

**Authors:** Saima Amin, Fawad Rizvi, Nida Zia, Amna Ali, Ahmer Hamid, Bhagwanti Kumari

**Affiliations:** 1 Department of Pediatric Ophthalmology, Layton Rehmatullah Benevolent Trust Tertiary Teaching Eye Hospital, Karachi, PAK; 2 Department of Vitreo-retina Ophthalmology, Layton Rehmatullah Benevolent Trust Tertiary Teaching Eye Hospital, Karachi, PAK; 3 Department of Pediatric Hematology and Oncology, The Indus Hospital, Karachi, PAK

**Keywords:** retinoblastoma group d, resistant seeds, diffuse vitreous seeds, melphalan, treatment outcomes

## Abstract

Introduction: A major therapeutic challenge in the salvage of Group D retinoblastoma eyes is the poor response of vitreous seeds to intravenous chemotherapy. The novel use of intravitreal melphalan has greatly impacted the salvage of such eyes; however, concerns regarding its safety and toxicity still exist, particularly in dark-eyed children. This study aims to evaluate our experience and determine the visual and anatomical outcomes of intravitreal melphalan in group D retinoblastoma with resistant vitreous seeds.

Method: All patients, from August 2018 to February 2020, with group D retinoblastoma harboring vitreous seeds refractory to first-line chemo reduction regimen with vincristine, etoposide, and carboplatin for six cycles plus local consolidation with thermotherapy or cryotherapy were evaluated. Fifteen eyes of 15 patients that fulfilled the eligibility criteria and received intravitreal melphalan were retrospectively reviewed for demographics, iris color, treatments offered, seed inactivation, globe survival, visual acuity, and complications.

Result: Mean age at presentation was 22 months for bilateral disease and 36 months for unilateral disease. A total of 77 injections were administered (mean, five injections per eye) with doses ranging from 20 µg to 30 µg. Complete seed control was seen in 13 of 15 (87%) eyes, and globe salvage was possible in 11 of 15 (73%) eyes. Eyes with macular tumor had visual acuity ranging from 6/36 (0.8) to 6/60 (1.0). SIx of eight eyes (75%) with extra macular tumors had vision 0.4 or better.

Conclusion: Intravitreal melphalan seems like a promising treatment modality in group D retinoblastoma with resistant vitreous seeds having dark eyes. Amblyopia therapy may play an important role in attaining maximal visual benefits in these children.

## Introduction

Retinoblastoma is the most encountered intraocular malignancy in children [1]. As with all cancers, the treatment of retinoblastoma depends upon the stage of the disease. To date, enucleation remains the safest treatment option for advanced cases; however, associated morbidity and parental lack of acceptance continue to be an issue of concern, particularly in bilateral cases. International Intraocular Retinoblastoma Classification (IIRC) of Group D retinoblastoma [2] corresponds to Reese Ellsworth group V which is labeled to carry the worst prognosis for globe salvage [3]. The prognosis was attributed partly to the poor control of vitreous seeds by systemic chemotherapy. The vitreous cavity is devoid of blood supply, and control of vitreous seeds relies mainly on the diffusion of a chemotherapeutic agent from retinal vessels [4]. The use of a chemotherapeutic agent targeted into the vitreous cavity was first encouraged by Suzuki et al., who concluded an eye salvage rate of 51% [5]; however, retinoblastoma cells have unstable chromosomes and are harvested at their highest mitotic index [6], causing them to infiltrate rapidly [7]. Therefore, intravitreal chemotherapy did not gain acceptance until Munier et al. developed an anti-reflux technique with needle track sterilization [8]. This novel method has shown to have minimal chances of extraocular seeding of tumor cells and very promising outcomes in globe salvage with advanced disease.

At our tertiary care eye hospital, we incorporated this treatment protocol, in coordination with the solid tumor division of the Pediatric Oncology department at a tertiary care facility, for improving outcomes in the management of retinoblastoma. In this retrospective study, we reviewed our experience with intravitreal melphalan in eyes classified as group D retinoblastoma (International Intraocular Retinoblastoma Classification [2]) for resistant vitreous seeds to assess anatomical and visual benefits in our local population over 18 months.

## Materials and methods

This study was approved by the institutional ethical review board. It was a retrospective review of the retinoblastoma database. All patients from August 2018 to February 2020, diagnosed with IIRC group D retinoblastoma at presentation with vitreous seeds refractory to systemic chemotherapy (carboplatin, vincristine, etoposide) six cycles were reviewed. Fifteen eyes of 15 patients who received intravitreal melphalan and fulfilled the following criteria were included in the study: (1) confirmation of absence of high-risk features (optic nerve involvement or choroidal involvement) on MRI; (2) absence of retinal detachment; (3) availability of tumor-free quadrant; (4) absence of tumor at the injection site; (5) absence of tumor at the entry site and at least two clock hours away; (6) absence of vitreous seed in the quadrant of entry; (7) absence of anterior segment invasion; (8) eyes classified as group D retinoblastoma (TNM 8th-cT2b). All patients who had partial treatment elsewhere or had less than six months follow-up were excluded from the study.

Data retrieved included patient's demographics, laterality, age at diagnosis, iris color, IIRC group, type of seeds (vitreous/subretinal), the distance of seeds from the tumor margin (in disc diameter), presence of retinal detachment, location of the tumor, location of seeds, number of cycles of chemotherapy given, type of focal therapy (laser or cryotherapy), complications recorded after intravitreal injection at each follow-up, eye salvage, recurrence, histopathology features where applicable, any evidence of metastases.

The main outcome measures were inactivation of vitreous seed (following any one of the regression patterns), globe salvage, and visual acuity.

All parents underwent detailed counseling regarding possible adverse effects of the injection and the possible outcomes of treatment. Consent was taken before each session, and the onco-pharmacy was informed regarding the dosage, which was calculated according to age as follows: 0-12 months: 20 micrograms, 1-3 years: 25 micrograms, and above 3 years: 30 micrograms.

**Figure 1:**
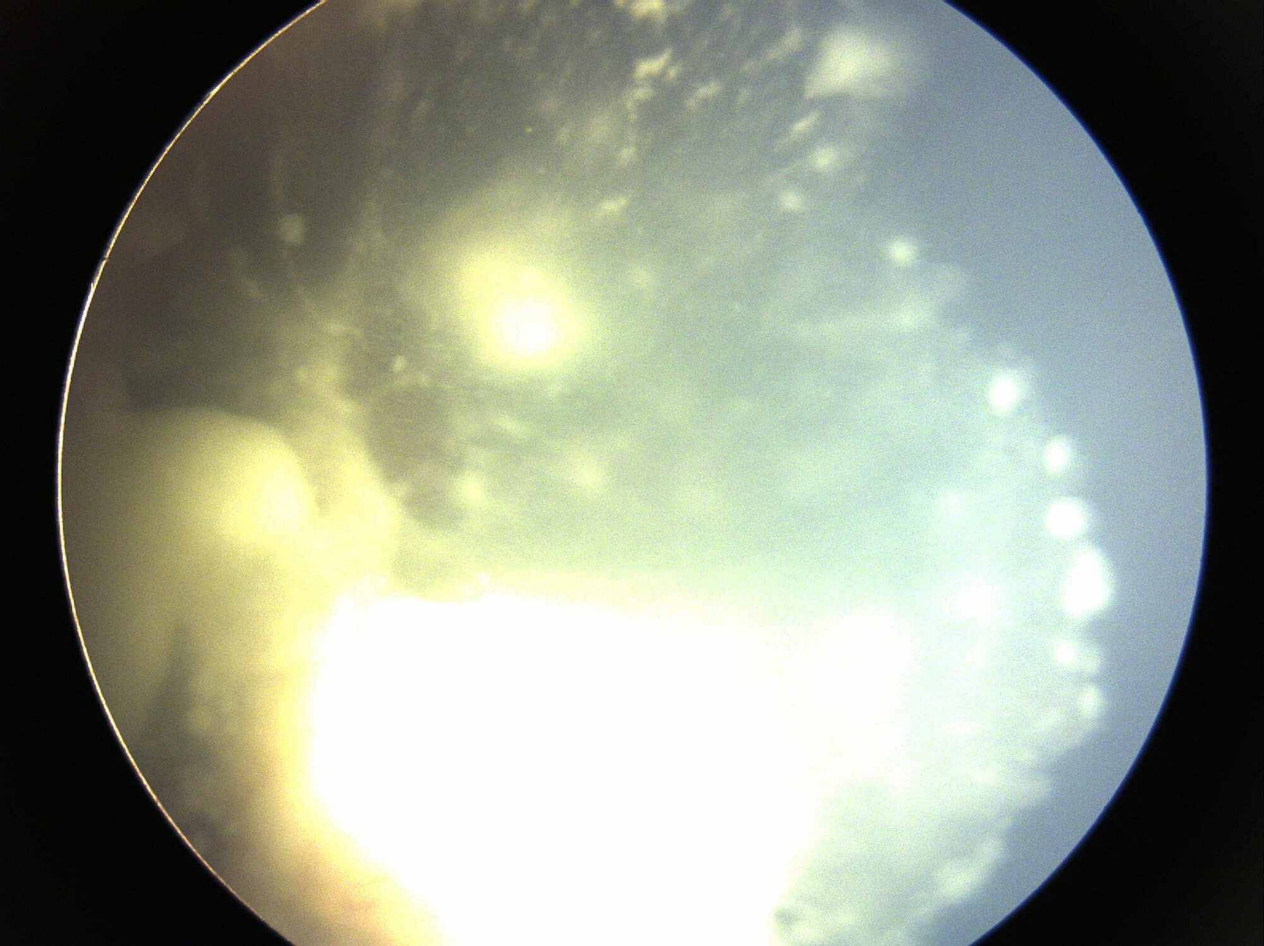
Group D retinoblastoma at presentation with extensive type 3 vitreous seeds (clouds)

**Figure 2:**
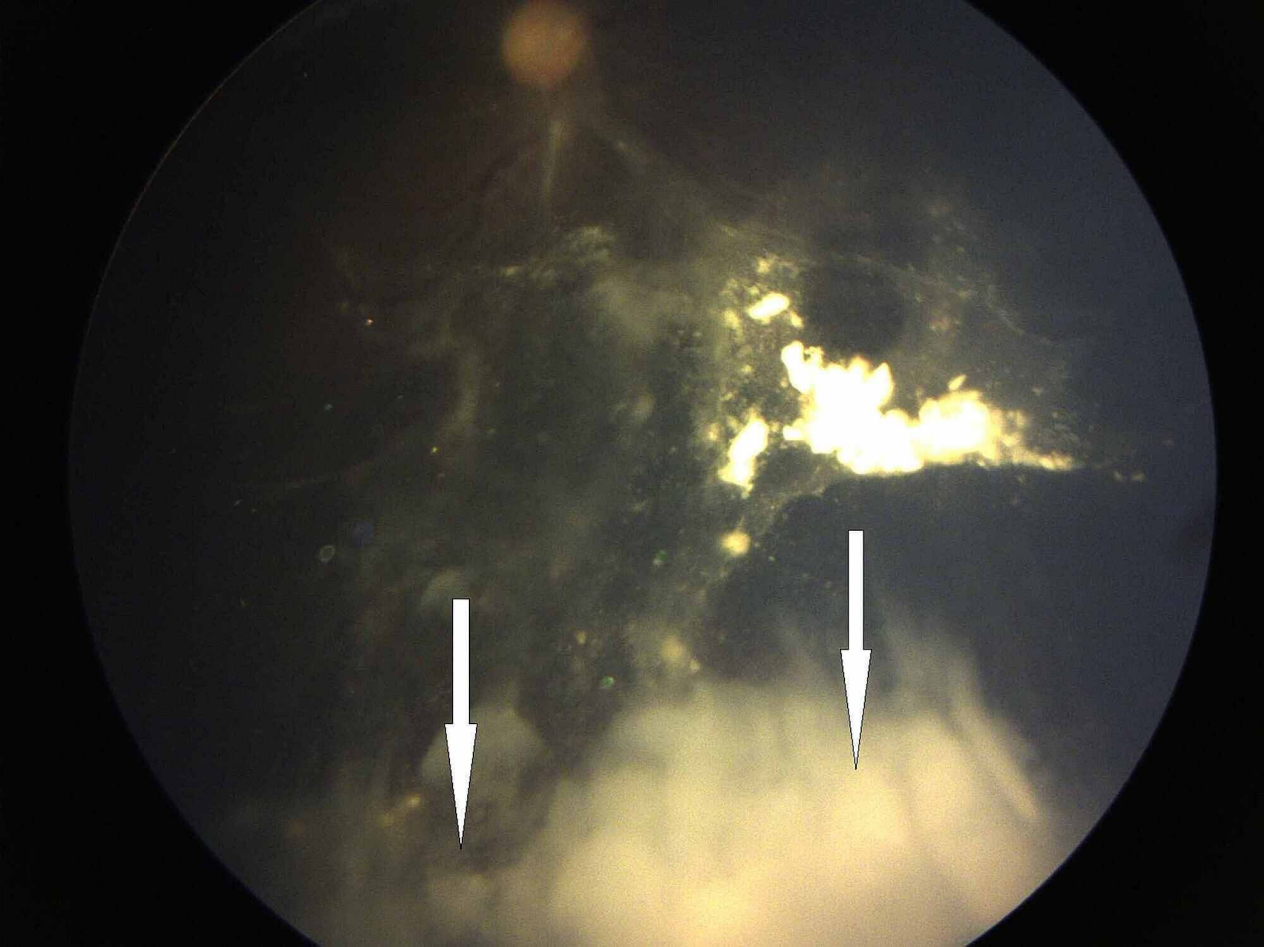
Resistant vitreous seeds type 3 (clouds) after four cycles of intravenous chemotherapy

**Figure 3:**
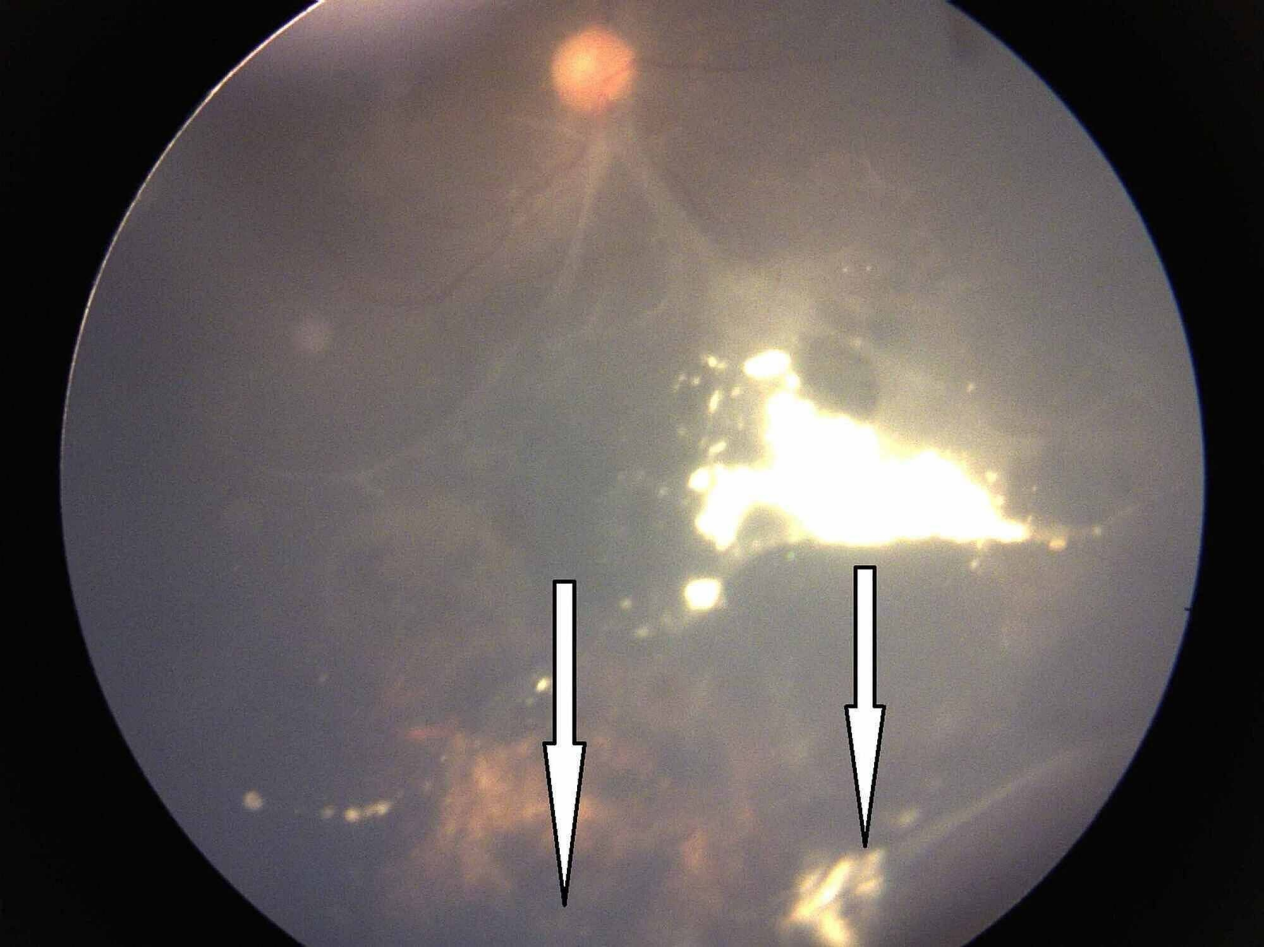
Group D retinoblastoma after five injections of intravitreal melphalan showing complete regression of vitreous seeds (Type 0)

Detailed examination under anesthesia (EUA) using an indirect ophthalmoscope, fundus photography using RetCam III (Clarity Medical Systems, Pleasanton, USA), and assessment of location, extent, nature, and response of vitreous seeds and solid tumors was noted. Vitreous seeds were defined as focal seeds if located within 3 mm of the tumor margin and diffuse if extended beyond 3 mm. The pattern of seeds was classified into type 1-dust, type 2-sphere, and type 3-clouds (Figures 1, 2). Regression response to treatment was classified into Type 0-complete disappearance of seeds (Figure 3), Type 1-calcific seeds, and Type 2-amorphous seeds. If required, focal treatment (laser and or cryotherapy) was applied before administering the injection.

Intravitreal injection technique

Cryo-machine was confirmed to be in working order before proceeding with the injection. Preoperative hypotony was created by applying firm pressure over the globe. A 30 gauge insulin syringe was used to inject the calculated dose. All patients received intravitreal melphalan (20-30 μg according to the age) by transconjunctival pars plana route 2.5-3.5 mm from limbus towards the vitreous cavity and directed away from the lens using a 30 gauge insulin syringe. Cryo-probe was applied immediately at the site of injection, and the needle was withdrawn from the ice-ball. Cryotherapy was then applied two more times at the injection site to kill any tumor cells that may have escaped, and the globe jiggled to distribute the drug within the vitreous cavity. The injection was repeated with a maximum of eight injections at an interval of 10 days.

Drug preparation and transportation

Several considerations regarding the preparation and transportation of melphalan hydrochloride are crucial for maximum drug efficacy. This drug is marketed as a 50 mg lyophilized powder and needs to be maintained at 2 degrees Celsius. It is transported to the onco-pharmacy unit, where it is reconstituted with preservative-free 0.9% sodium chloride solution in a sterile chamber. The drug should be administered within 15 min and not more than 60 min after reconstitution since diluted melphalan is unstable and undergoes spontaneous hydrolysis to monohydroxymelphalan [9]. The onco-pharmacist was requested to inform immediately as the drug reconstitution was started to ensure that the child is prepared under anesthesia and the surgeon is draped and ready.

The response was categorized as complete, partial, or failure. Complete was defined as complete inactivation of vitreous seeds following any one of the regression patterns. Partial was defined as at least 50% inactivation of vitreous seeds. Failure was defined as eyes with any of the following response: (a) less than 50% regression, (b) recurrence of new seeds during the treatment or within three months of discontinuation of treatment, (c) development of any complication of intravitreal melphalan requiring enucleation, or (d) evidence of local or distant metastasis. Follow-up was advised every four weeks for the initial three months, then extended if no active lesion was seen. Therapeutic success was defined as a stable regression of all seeds without recurrence. Two-tailed Fisher's exact test was used to calculate the p-value.

Recording of visual acuity

Visual acuity (VA) was measured monocular with the best refractive correction in place before each EUA. Sine gratings were used for children less than 18 months. For older children, crowded kay pictures were used. The VA was recorded in logMAR units for the number of optotypes the child identified correctly. All children were strictly advised patching therapy if the fellow eye was better for four to six hours, depending on the visual status and response to treatment.

Clinical characteristics and definitions

Group D eyes were defined as eyes having at least one of the following features: diffuse vitreous, or subretinal seeds, massive non-discrete endophytic or exophytic disease, exudative retinal detachment involving more than one quadrant of the retina, or tumor involving >50% of the globe.

Diffuse vitreous seeds were defined as vitreous seeds located at least 3 mm from the tumor.

Macula was defined as area of the retina within a radius of 3 mm from the fovea.

## Results

Twenty eyes of 20 patients that received intravitreal melphalan were identified. Five were lost to follow-up either due to lack of accommodation or due to their belief in alternative medicine. Fifteen eyes of 15 patients that fulfilled the study criteria were included. All children were black-eyed. The total number of injections administered was 78, with a mean of five injections per eye, and the dose was adjusted according to age. The mean age at presentation was 22 months for bilateral disease and 36 months for unilateral disease. There were seven (47%) males and eight (53%) females.

Disease characteristics

Nine (60%) children had bilateral disease, and six (40%) had unilateral disease. In bilateral disease, the fellow eye was enucleated in five (56%) children. The main tumor was located anteriorly in seven (47%) eyes, posteriorly in five (33%) eyes, and dispersed in three (20%) eyes.

In bilateral disease, five of nine (56%) children had undergone fellow eye enucleation. According to vitreous seed classification, predominantly type 1 seed was found in three (20%) eyes, type 2 in five (33%) eyes, type 3 in seven (47%) eyes. In all cases, the disease was diffuse, involving more than one quadrant of the eye.

Response of vitreous seeds

Overall complete regression of vitreous seeds was seen in 13 eyes (87%). This has been categorically displayed in Table 1.

**Table 1:** Correlation between type of seed and response of intravitreal melphalan

Type of seed	Complete response (n=13)	Partial response (n=2)	P=1.00
Type 1 (dust)	3 (23%)		
Type 2 (sphere)	4 (31%)	1 (50%)	
Type 3 (cloud)	6 (46%)	1 (50%)	

Vitreous seed regression was seen as complete disappearance (Type 0) in seven eyes (47%), calcific seeds (Type 1) in six eyes (40%), and amorphous seeds (Type 2) in two (13%) eyes. The median number of injections needed to get a complete response was three injections for dust, four injections for sphere, and six injections for clouds (Table 2).

**Table 2:** Correlation of the type of seed to the required number of injections

No of injections	Seed type 1	Seed type 2	Seed type 3	total	P=0.017
3	2	1	0	3	
4	0	1	0	1	
5	0	1	4	5	
6	0	0	4	4	
8	0	0	2	2	
total	2	3	10	15	

Outcome of eyes

The median follow-up time after completion of treatment was 18 months (3-48 months). Two of 15 (13%) eyes showed only partial regression with the development of new subretinal seeds and were enucleated. Both eyes showed low-risk histopathological features. Out of 13 eyes that showed complete regression, one eye developed tractional retinal detachment due to extensive retinal fibrosis and tumor recurrence two months after completion of treatment. Parents were advised enucleation. The decision was delayed by four months. Histopathology revealed the presence of intermediate risk (anterior segment invasion and post laminar invasion but not to cut end). The other eye underwent enucleation after the recurrence of the main tumor with overlying vitreous seeds nine weeks after discontinuation of melphalan. No evidence of high-risk features on histopathology was seen in this eye.

The most common adverse effect was pigmentary retinopathy in eight (53%) of 15 eyes. Overall, 11 eyes (73%), otherwise scheduled for enucleation, were salvaged with intravitreal chemotherapy.

The most common adverse effects encountered were pigmentary retinopathy and posterior synechiae (Table 3). Anterior uveitis was mild, transient, and self-limited.

**Table 3:** Adverse effects of intravitreal melphalan

Adverse Effects	Number of Cases
Cataract	1 (6%)
Pigmentary retinopathy	8 (53%)
Vitreous fibrosis	5 (33%)
Tractional retinal detachment	1 (6%)
High risk feature on histopathology	1 (6%)
Posterior synechiae	8 (53%)
Anterior uveitis	5 (33%)

Visual acuity

Following up at six months, the final visual acuity after completion of treatment in eyes that were salvaged was found to be at least 6/60 (logMar 1.0) or better in all children. Six (54%) of children had vision 6/24 (logMar 0.6) or better. Eyes with macular tumors had visual acuity ranging from 6/36 (0.8) to 6/60 (1.0). Six of eight eyes (75%) with extra macular tumors had vision 0.4 or better. No association was found between the number of injections and visual acuity (P=0.86).

## Discussion

Group D retinoblastoma outlines features of tumor dissemination. Dispersed vitreous seeds arising from active tumors may be a source of tumor implantation at other sites and hinder visualization and access of focal therapy to the main tumor. Active vitreous seeds resistant to systemic chemotherapy are a major cause of treatment failure in group D tumors owing to insufficient chemotherapeutic levels within the vitreous cavity [10]. In this series, we retrospectively analyzed the response and outcomes of group D eyes that harbored resistant vitreous seeds and were treated with intravitreal Melphalan with a minimum follow-up of six months.

It is seen that the response to intravitreal melphalan is dose-dependent. The dose of intravitreal melphalan used in this study was between 20-30 µg; although higher and lower doses have also been studied [5,11,12], this seems to be the most widely adopted dose range for better clinical benefit and accepted levels of toxicity [10]. In this study, we found that there was at least partial response in vitreous seed regression in 100% of eyes; however, the success rate of intravitreal melphalan in seed control was seen in 13 of 15 eyes (87%). Overall, globe salvage was possible in 11 eyes (73%) due to the development of new, uncontrollable subretinal seeds in two cases at a minimum follow-up of six months (6 months-18 months). Shield et al. reported a success rate of 100% in a preliminary report on 11 eyes with 0% recurrence at nine months with a similar dose, but included eyes with group C and D retinoblastoma and also used a combination of topotecan and melphalan [13]. A study conducted by Ji et al. in China included 19 eyes with focal and diffuse seeds and showed a success rate of 84% using 20 µg [14]. Similarly, Yousef et al. recently reported a success rate of 81% with a similar dosage regimen and included all eyes with vitreous seeds except group E [15]. We faced two cases of recurrence after completion of treatment, which decreased the overall globe survival rate. Most studies included all eyes with focal and diffuse seeds regardless of the IIRC group at presentation, which may carry different prognoses [2].

The number of injections required for seed control depends on the response to treatment. Studies have reported having administered up to 25 injections [5,14], but most studies report six to eight injections at dose range of 20-30 µg [15-17]. Our study shows that the number of injections required for seed control was associated with the density of vitreous seeds (p=0.01). The mean number of injections administered in this study was two, three, and six for vitreous seeds type 1, 2, and 3, respectively. A similar pattern has been seen in studies using the same dosage regimen [17,18]. Yousef et al. reported the highest number of injections for vitreous clouds and mentioned the need for cloud dispersion for active tumor cells to be exposed to the drug as a plausible explanation [15]. Francis et al. also highlighted the possible need to titrate the cumulative dose according to the density of vitreous seeds [16].

Despite the staggering benefits of intravitreal melphalan for seed control, concern still exists regarding its toxicity. Francis et al. investigated toxicity in 130 eyes and elucidated the likelihood of exaggerated toxicity in darkly pigmented eyes due to the increased accumulation of the drug seen at the level of retinal pigment epithelium and choroid [19]. Based on another theory [20] that darkly pigmented eyes are more vulnerable to intraocular inflammation. Xue et al. also postulated that intravitreal melphalan in the Asian population might incite inflammation more than that of the Caucasian population [21]. Some studies support the use of topotecan in such eyes as it is thought to induce lesser inflammation [22,23]. In this study, only transient mild anterior uveitis was noted in five cases (33%), but posterior synechia was found in 53% of eyes after a minimum of three injections. However, it is to note that all these eyes were concurrently undergoing focal therapy (laser 532 green laser and/or cryotherapy) for solid tumor control, which could also be responsible for the inflammation. Posterior subcapsular cataract was seen in one eye (6%). No other sign of uveitis was noticed in any eye. Localized pigmentary retinopathy (salt and pepper fundus) was also seen as a common side effect in nine of 11 eyes (53%) only in the vicinity of the injection site. A plausible explanation of this finding mentioned in literature is the exposure of a high concentration of the drug as it is introduced. However, it is believed that globe jiggling enhances the uniform spread of the drug in the vitreous cavity [8]. A high-risk histopathology feature was seen in one case with a massive recurrence of subretinal seeds and new active tumors. This was most likely due to a delay in enucleation.

A study conducted by Francis et al. on the toxicity of intravitreal melphalan shows a reduction in electroretinogram responses, which may further be exaggerated in darkly pigmented eyes [19] however, the extent to which it translates to a reduction in visual acuity is yet to be determined. In our series, we initiated patching therapy early in the course of treatment in all children in whom the fellow eye was normal. Seventy-five percent (6/8) eyes with extra macular tumor location were found to have a vision of 0.4 or better. The median number of injections administered in these eyes was five (3-8 injections, mean=5). The critical period in binocular visual development begins from a few weeks after birth till 5-7 years of age, after which the cortical plasticity decreases [24]. Asymmetry in visual signals during this period is highly amblyogenic, which coincides with the manifestation of the disease itself. Therefore the role of patching in the better eye early in the course of treatment may play a crucial role in attaining maximal visual benefit.

Vitreous seeding is the most dreadful complication in the eye receiving intravitreal injections. The anti-reflux technique introduced by Munier et al was a breakthrough in the management of an eye laden with vitreous seeds. As promising as it seemed, it was quickly adopted by most retinoblastoma centers across the globe, and published data shows promising results to date [11,13,14,16]. In his report, he highlighted key measures to minimize any possibility of extraocular spread and conducted a study on 30 eyes that received 135 injections with no evidence of extraocular seeding at a median follow-up duration of 13.5 months [8]. Similar outcomes were later reported by Ghassemi and Shields [11,13]. In our series, we adopted similar protocols: 1) strict adherence to the eligibility criteria mentioned above, 2) choosing the quadrant devoid of vitreous seeds, 3) creating preoperative hypotony by applying firm pressure on the globe, 4) creating an ice ball around the needle before withdrawal, and 5) triple freeze-thaw over the injection site. Out of 15 eyes, we did not experience any case of orbital seeding at a minimum of six months follow-up (6 months-18 months).

## Conclusions

We conclude that intravitreal melphalan had promising outcomes in this series of dark-eyed children with resistant disseminated vitreous seeds, in terms of anatomical and visual benefit. We emphasize the need for early patching if the fellow eye has better visual status. A major issue of parental delay in acceptance of enucleation in a case that mandated urgent intervention was seen. The prognosis of advanced retinoblastoma is poor and the outcome is multi-factorial, instituting salvage therapy may make parents over-optimistic regarding eye salvage, therefore, parents need to be properly counseled regarding the possible outcomes. 
